# Antineoplastic effects of sodium dichloroacetate and omeprazole, alone or in combination, on canine oral mucosal melanoma cells

**DOI:** 10.3389/fvets.2023.1186650

**Published:** 2023-07-13

**Authors:** Gabriela F. Toledo, Marcia K. Nagamine, Victor Nowosh, Felippe T. Machado, Cristina O. Massoco, Nadja C. Souza-Pinto, Maria L. Z. Dagli

**Affiliations:** ^1^Laboratory of Experimental and Comparative Oncology, School of Veterinary Medicine and Animal Science of the University of São Paulo, São Paulo, Brazil; ^2^Laboratory of Comparative Imuno-Oncology, School of Veterinary Medicine and Animal Science of the University of São Paulo, São Paulo, Brazil; ^3^Laboratory of Mitochondrial Genetics, Department of Biochemistry, Institute of Chemistry, University of São Paulo, São Paulo, Brazil

**Keywords:** canine melanoma, DCA, omeprazole, drug repurposing, synergism

## Abstract

Oral mucosal melanoma (OMM) is a common neoplasm in canines, although it is rare in humans. Cancer cells present alterations in energetic metabolism, and the Warburg effect states that most cancer cells undergo aerobic glycolysis. This can be reversed by certain drugs, resulting in decreased cell viability and cell death. We sought to evaluate the effects of sodium dichloroacetate (DCA) and omeprazole (OMP) alone or in combination on canine OMM and human melanoma cells. CMGD5 and SK-MEL-28 cell lines were treated with DCA and OMP alone or in combination, and cell viability was assessed using the crystal violet assay. Cell death (apoptosis and necrosis) was assessed by Annexin V and propidium iodide (PI) staining assays using flow cytometry. In addition, the oxygen consumption rate (OCR) was evaluated using a SeaHorse XF assay. Treatment with DCA or OMP alone resulted in a significant, but not dose-dependent, reduction in cell viability in both cell lines; however, the combination of DCA and OMP resulted in a significant and dose-dependent decrease in viability in both cell lines. DCA and OMP, alone or in combination, did not alter OCR at the concentrations tested in either cell line. Since the combination of DCA and OMP potentialized the inhibition of viability and increased cell death in a synergistic manner in melanoma cells, this approach may represent a new repurposing strategy to treat cancer.

## 1. Introduction

Oral mucosal melanoma (OMM) is the most common malignant tumor of the oral cavity in dogs, but is rare in humans. OMM is well known for its aggressive behavior and resistance to conventional treatment. Treatment options for dogs with OMM include radical surgical resection of the tumor and regional lymph nodes, radiation therapy, chemotherapy, and immunotherapy depending on the staging of the tumor. However, many dogs develop metastatic disease and die within to 8–24 months of diagnosis and treatment. Owing to the poor prognosis and outcomes associated with conventional treatments for canine patients with oral melanoma, it is of great importance to investigate novel, efficient, and affordable treatment options. It is known that OMM is highly aggressive such in dogs as in humans, and the canine OMM is considered a suitable model for the human disease (1–3).

As it is well established, cancer cells develop many different metabolic adaptations to survive and maintain carcinogenesis in a hostile environment. Because of this knowledge, metabolic pathways in malignant tumors have become one of the most studied hallmarks of cancer in recent decades alongside with other capabilities such as the cancer cell ability to unlock phenotypic plasticity to evade and escape cell-differentiation (4, 5). The Warburg effect describes the metabolic process of energy production by most cancer cells, and relies on aerobic glycolysis even in the presence of oxygen, providing less ATP per glucose molecule oxidized and higher rates of lactate production, thus granting many beneficial characteristics for tumor progression, such as providing nutrients and biosynthesis for rapid mitotic rates, activating oncogenes and growth factors, resisting cell death, evading immunosurveillance, and promoting drug resistance (4, 6, 7).

Dichloroacetate sodium (DCA) is a small, low-cost molecule that exerts antineoplastic effects due to metabolic reprogramming in many solid tumors. It is a pyruvate dehydrogenase kinase (PDK) inhibitor that promotes aerobic glycolysis in many malignant solid tumors. In cancer cells, DCA acts as an “metabolic modulator” redirecting the metabolism of aerobic glycolysis (Warburg effect) to mitochondrial phosphorylation, drastically reducing the production of lactate and protons and redirecting energy production to oxidative phosphorylation. It has a dual apoptosis mechanism: depolarizing mitochondria and upregulating Kv.1.5 channels, thus targeting cancerous cell apoptotic mechanisms (8–10).

Owing to the inhibition of Pyruvate dehydrogenase (PDH) cancerous cells tend to convert much more lactate in the cytoplasm. A normal cell does not survive with the subsequent over-accumulation of intracytoplasmic protons; however, cancer cells make use of many adaptations to survive in acidic microenvironments. One of the most important phenotypic changes is the upregulation of proton pumps (PPs) and proton transporters (PT) in cell membranes, which actively pump protons from the cytosol to the extracellular membrane. This very important survival adaptation to pH acidification in the surrounding tumor microenvironment has been related to drug resistance, metastasis, and progression of different tumors (11, 12). In addition, higher levels of, lactate generated by tumors inhibit T cell export and dendritic cell activation, inhibit monocyte migration and cytokine release, and enhance tumor motility and dissemination (13).

Omeprazole (OMP) is a well-established, low-cost prodrug that belongs to the proton pump inhibitor (PPI) class and is used worldwide as a standard drug for the treatment of gastric and duodenal ulcers, gastroesophageal reflux, and as a gastrointestinal protectant in both veterinary and human medicine (14, 15). In cancer, it seems to be activated in the presence of the acidic tumor microenvironment (15, 16). However, its mechanisms of action are still under investigation. Studies have proposed that one of the main mechanisms of action of OMP in cancer is the inhibition of V-ATPase, which neutralizes pH and signals pro-apoptotic factors (11, 12, 16, 17).

Evidence-based studies have demonstrated that the use of DCA as a main form of treatment provided a long-term stabilization of metastatic melanoma in human patients for >4 years (18). A recent study showed that DCA markedly inhibits the proliferation and viability of lung cancer colonies *in vitro* and enhances the effects of the chemotherapeutic agent cisplatin on these cells (19). OMP has been shown to inhibit proliferation of pancreatic cell lines (20). In a phase I/II clinical trial, 34 companion animals with spontaneous tumors were treated with high doses of Lansoprazole, a PPI in the same family as OMP, associated with conventional chemotherapy which resulted in a positive outcome for most patients treated with the combined treatment, either presenting partial or complete treatment responses (21).

Owing to these remarkable antineoplastic effects on various malignant cancers, we hypothesized that the two drugs may act synergistically as antineoplastic compounds in canine OMM and compared their effects on human cutaneous counterparts.

## 2. Materials and methods

### 2.1. Ethics

The study protocol was approved by the Committee on Ethics on the Use of Animals (CEUA) of the School of Veterinary Medicine and Animal Science of the University of São Paulo, Brazil (Process number 8292290119).

### 2.2. Melanoma cell lines

The CMGD5 canine oral melanoma cell line was acquired from Kerafast (Boston, USA); the SK-MEL-28 human cutaneous cell line was acquired from Banco de Células do Rio de Janeiro (RJ, Brazil). Cell lines were cultured in cell culture flasks with Dulbecco's Modified Eagle's Medium (DMEM) high glucose (Thermofisher—Gibco, USA) supplemented with 10% fetal bovine serum (FBS, Thermofisher—Gibco, USA) and 1% antibiotic-antimycotic (Thermofisher—Gibco, USA) in a 37°C 5% CO_2_ humidified incubator. The cells were routinely tested for mycoplasma contamination using qPCR.

### 2.3. Cell viability assay

Cells from each lineage were seeded into 96-well plates and divided into four different groups containing 4 replicates: (1) DCA treatment; (2) OMP treatment; (3) DCA and OMP combination; and 4) control group. The drug concentrations used in this assay ranged from 2.5 to 40 mM for DCA and 44.44 to 225 μM for OMP. The control group was treated with dimethyl sulfoxide (DMSO; vehicle for OMP) or cell culture medium (vehicle for DCA). The cell lines were treated with different concentrations of DCA and OMP alone or in combination for 48 h. The control group was also treated for the same period. Cytotoxic effects were evaluated by crystal violet colorimetric assay, in which the remaining adherent cells after treatment were stained with 0.5% crystal violet and the dye solubilized in methanol was read in a spectrophotometer at 570 nm. Optical density (OD) values were transformed into percentages of relative cell viability by dividing the OD values of the drug treatments by the OD value of the vehicle control.

### 2.4. Synergy assay

The synergistic drug combinations were evaluated using the SynergyFinder software version 3.0 (https://synergyfinder.fimm.fi) using the ZIP (Zero interaction potency) model. The data for analysis were the same as for the crystal violet assay. The synergy interaction will generate a score which can be interpreted as follows: < -10: likely antagonistic; from −10 to 10: likely additive; and >10: likely synergistic. The grade of interaction between the two drugs is shown in a 2D graph, which will differ the regions of synergistic interactions in red and the antagonistic regions in green. The dose-response matrix map will also exhibit the level of interaction of both drugs, according to the concentration of both drugs and its inhibition percentage. High inhibition percentage will appear vivid red on the map, as for the lowest inhibition concentrations will appear orange or in lighter colors.

### 2.5. Cell death analysis using flow cytometry

CMGD5 and SK-MEL-28 cells (6 × 10^4^ cells/well) were seeded in 6-well sterile plates in triplicates for each group of treatment. After 24 h of cellular adhesion to the plates, the cells were exposed to different concentrations of the drug combinations in a single-dose treatment, then incubated for 72 h. The control group was treated with 0.1% v/v DMSO. Drug concentrations were established according to the literature and previous experiments. DCA and OMP were administered in the following groups: 10 mM DCA + 100 μM OMP; 10 mM DCA + 200 μM OMP; 20 mM DCA + 100 μM OMP; and 20 mM DCA + 200 μM OMP.

After 72 h of incubation, the cells were harvested from 6-well plates by trypsinization and washed with phosphate buffered saline (PBS). The cells were centrifuged at 392 g for 5 min and the supernatant was discarded. Next, the Alexa Fluor 488/Annexin V/Dead Cell Kit (Invitrogen, USA) was used for the staining protocol before analysis by flow cytometry following the manufacturer's instructions. Apoptosis and necrosis are distinguished in cells mainly by modified morphology. In apoptotic cells, phosphatidylserine (PS) is located on the outer leaflet of the cell membrane. Because annexin V has a high affinity for PS, annexin V is labeled with a highly sensitive fluorophore (Alexa Fluor 488), which provides green light in apoptotic cells. Propidium iodide is a nucleic acid binding dye that cannot permeate apoptotic or live cells but binds directly to nucleic acids of dead cells, providing a red-fluorescent spectrum to cells that undergo necrosis. After the staining procedure, each group of cells was immediately analyzed using a BD FACSCalibur flow cytometer (BD Biosciences, USA).

### 2.6. Analysis of oxygen consumption rate in intact cells

To determine whether DCA and OMP act as metabolic modulators in mitochondria, CMGD5 and SK-MEL-28 cell lines were seeded at 3 × 10^3^ cells/well in a SeaHorse 24-well cell culture plate. After 24 h of cell adhesion, the cultures were treated in quadruplicate with different concentrations of DCA and OMP as follows: (1) DCA 10 mM; (2) OMP 100 μM; (3) DCA 10 mM + OMP 100 μM; and (4) vehicle control group (0.1% v/v DMSO). After drug addition, the plates were incubated under standard conditions for 24 h. The next day, the treatments were removed, the cultures were washed with PBS, and the medium was replaced with Seahorse XF base-adapted medium (Dulbecco's Modified Eagle's medium without sodium bicarbonate or bovine fetal serum, pH 7.4), followed by incubation at 37°C in a non-CO_2_ incubator for 1 h. To evaluate mitochondrial respiration, the following metabolic inhibitors were used: oligomycin (inhibitor of ATP synthase, 1 μM), carbonyl cyanide-p-trifluoromethoxyphenylhydrazone (FCCP, uncoupler of mitochondrial oxidative phosphorylation, 1.5 μM), a combination of rotenone (mitochondrial complex I inhibitor, 0.5 μM), and antimycin A (complex III inhibitor, 0.5 μM). Three basal OCR measurements were made before any addition; after each addition, and three additional measurements were taken before the next addition. Three independent experiments were performed. The OCR was automatically recorded and calculated using Seahorse XFe96 software (Wave 2.3.0). To normalize the OCR to the cellular density in each well, after the measurements, the medium was removed and the cultures were washed with PBS and fixed with 10% Trichloroacetic Acid. To each well, 200 μl of 1 M NaOH was added, and the plates were incubated at 37°C for 1 h. Cell density was estimated by measuring the optical absorbance at 260 nm in a plate reader. The OCR for each well was normalized to A_260_ for the corresponding well before any further calculation.

### 2.7. Statistical analysis

Statistical analysis of the treatment groups in comparison to control groups in crystal violet assay was performed using one-way ANOVA followed by Dunnett's post-test. Annexin assay was evaluated by two-way ANOVA followed by Bonferroni's post-test. OCR was evaluated using the Kruskal Wallis test. Statistical significance was set at *p* < 0.05.

## 3. Results

### 3.1. Combination of DCA and OMP revealed concentration-dependent cytotoxicity

The effects of DCA and OMP alone on the viability of CMGD5 and SK-MEL-28 cells were investigated using the Cristal Violet Assay, as described in the text.

**Figure 1:**
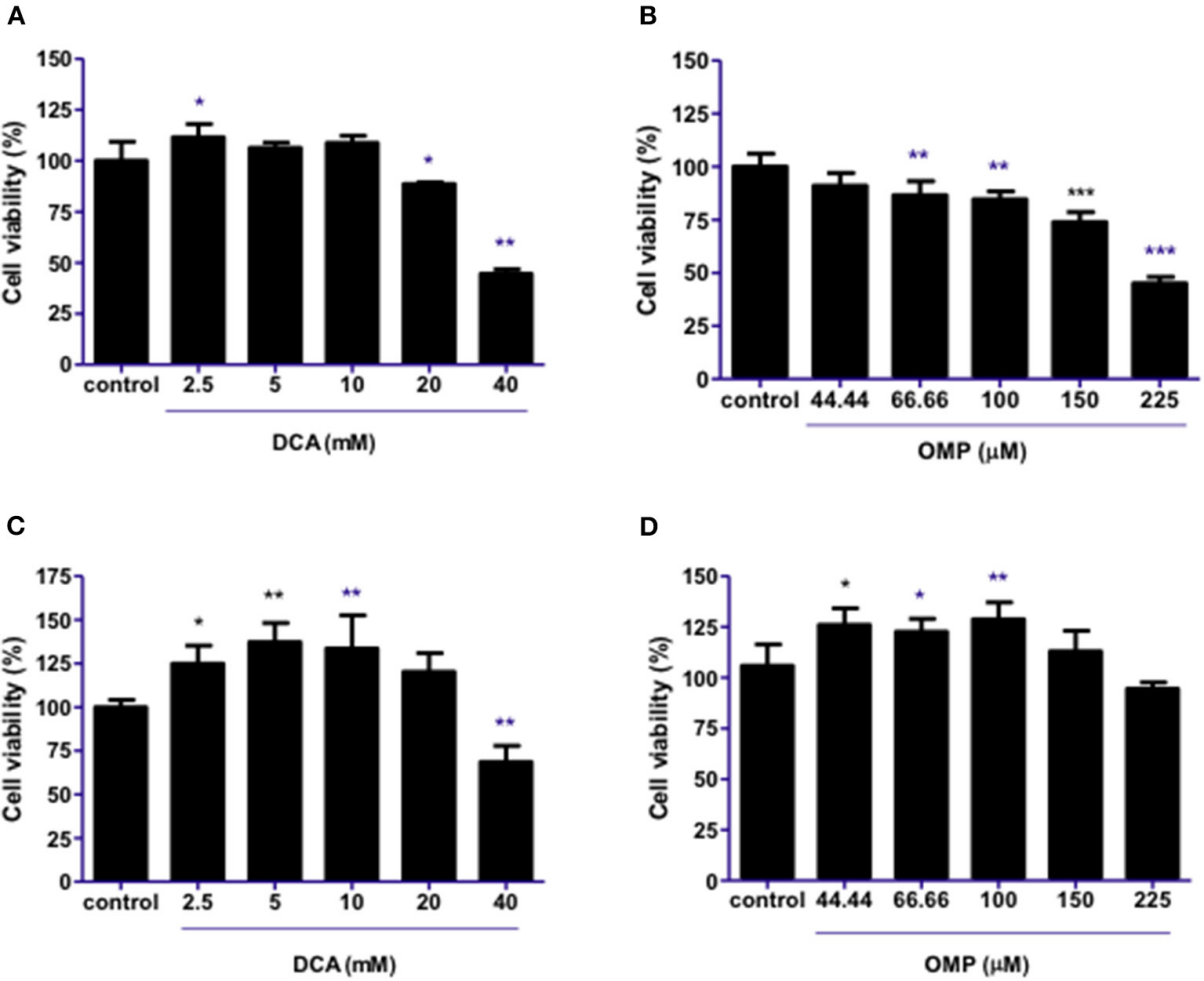
DCA and OMP effects on cell viability of canine oral melanoma cell line CMGD5 **(A, B)** and cutaneous human melanoma SK-MEL-28 **(C, D)**. Cells were treated with single drugs at different concentrations and evaluated after 48 h using a crystal violet assay. **(A, C)** DCA treatment (2.5–40mM). **(B, D)** OMP treatment (44.44-225 μM). *p < 0.05; **p < 0.1; ***p < 0.001.

DCA produced a hormesis effect when used alone in CMGD5 canine oral melanoma cells due to its response on cell viability at lower concentrations such as 2.5–10 mM, and inhibition at higher concentrations [20 and 40 mM (Figure 1A)]. As for OMP treatment, CMGD5 cells responded with inhibition of viability beginning at a concentration of 66.66 μM in a dose-dependent manner (Figure 1B). The assay was replicated to observe the effects of DCA and OMP alone on the human cutaneous melanoma cell line SK-MEL-28. Unlike the CMGD5 cell lineage, SK-MEL-28 cells were resistant to DCA treatment, exhibiting an inhibiton effect only at the highest concentration of 40 mM (Figure 1C). SK-MEL-28 cells showed no inhibitory effects after treatment with OMP at any concentration; however, a hormetic effect was also observed (Figure 1D).

**Figure 2:**
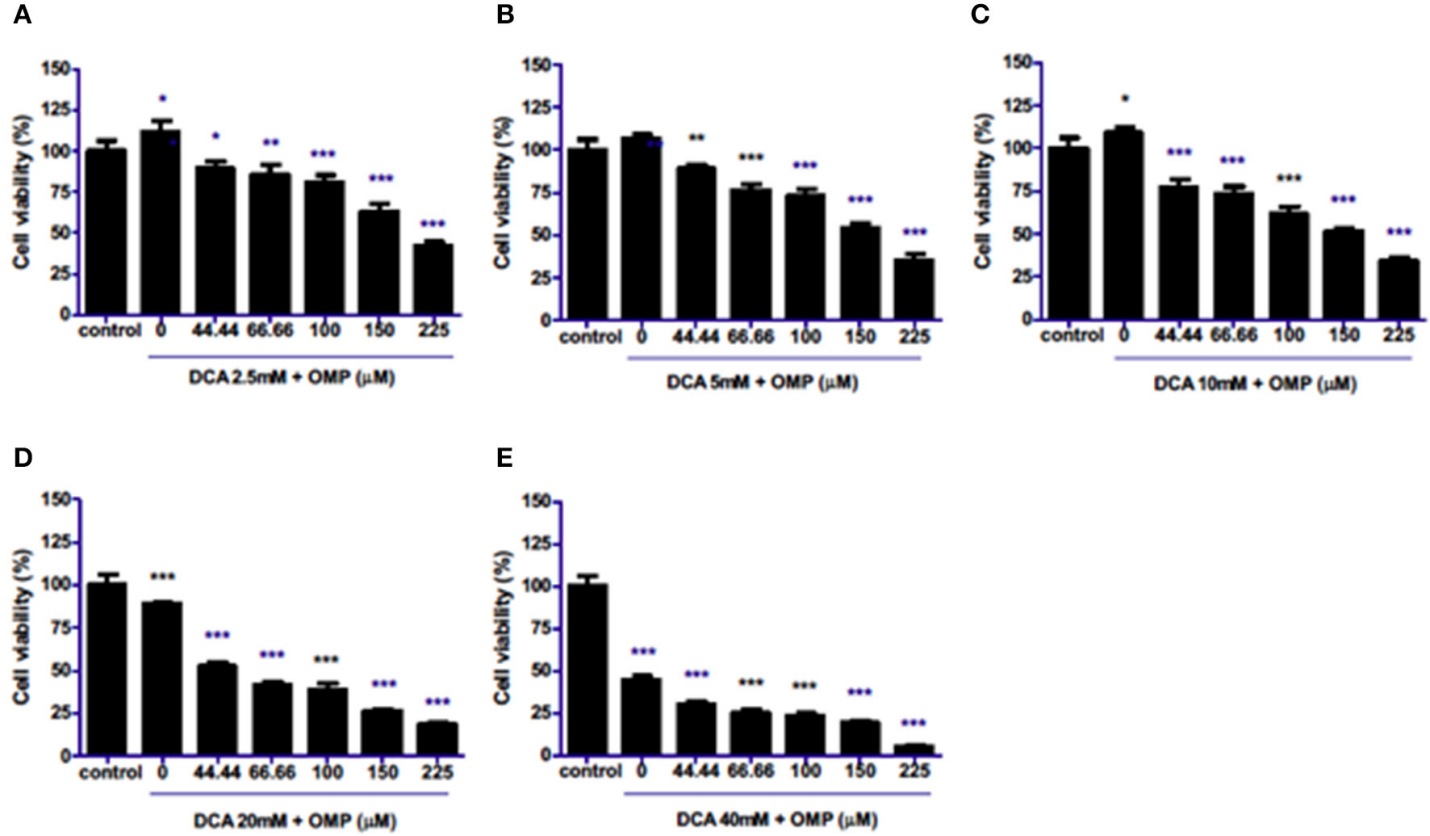
Co-treatment of CMGD5 cells with DCA and OMP. **(A–E)** Different combinations of DCA (2.5–40mM) and OMP (44.44–225 μM) exhibit a decreasing effect in the percentage of cell viability in the crystal violet assay. *p < 0.05; **p < 0.01; ***p < 0.001.

The effects of combination therapy with both drugs on the same cell lineages were evaluated. Surprisingly, the drugs synergistically inhibited the viability of cells in a concentration-dependent manner at all concentrations (Figures 2A–E) in CMGD5 cell line.

**Figure 3:**
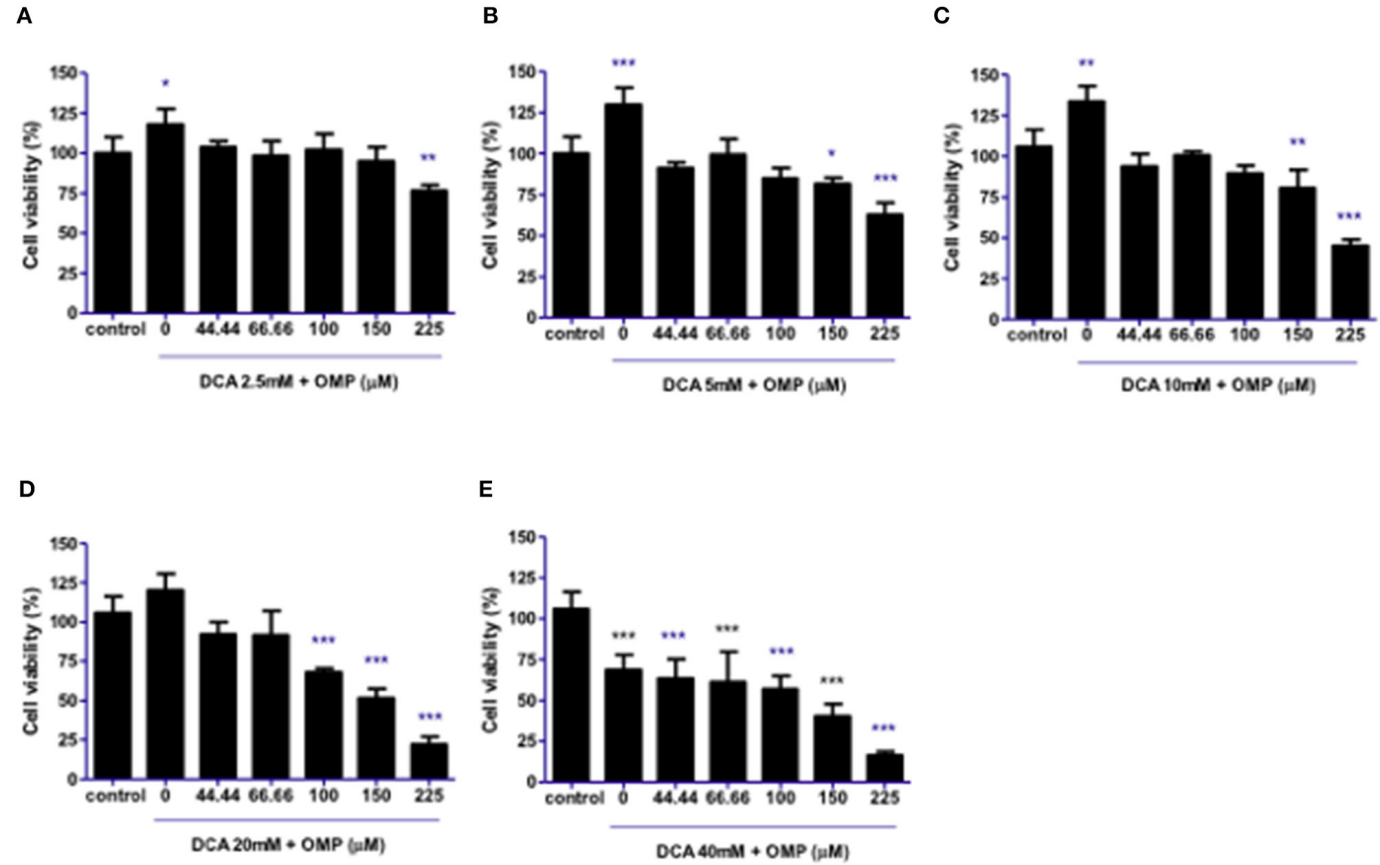
Co-treatment of cutaneous human melanoma cell line SK-MEL-28 with DCA and OMP. **(A–E)** Combined treatment of DCA (2.5–40mM) and OMP (44.44–225 μM) showing a decreasing effect in the percentage of viable cells in the crystal violet assay. *p < 0.05; **p < 0.01; ***p < 0.001.

This study was repeated to observe the effects of DCA and OMP on the human cutaneous melanoma cell line SK-MEL-28. In contrast to CMGD5 cells, SK-MEL-28 cells were slightly more resistant to the combined treatment of DCA and OMP, and did not present inhibitory effects at the lowest combination of DCA and OMP (Figure 3A), except at higher dose of OMP. Treatment with 5 mM and 10 mM of DCA showed decrease in cell viability only in the highest concentrations of OMP (Figures 3B, C), while 20 mM DCA starts at 100 μM OMP (Figure 3D). The higher concentration of DCA shows inhibitory effect in all associations with OMP (Figure 3E).

### 3.2. Combination of DCA and OMP revealed synergistic effects

To evaluate the interaction between DCA and OMP, the data obtained in the crystal violet assay were analyzed using the web-application SynergyFinder.

**Figure 4:**
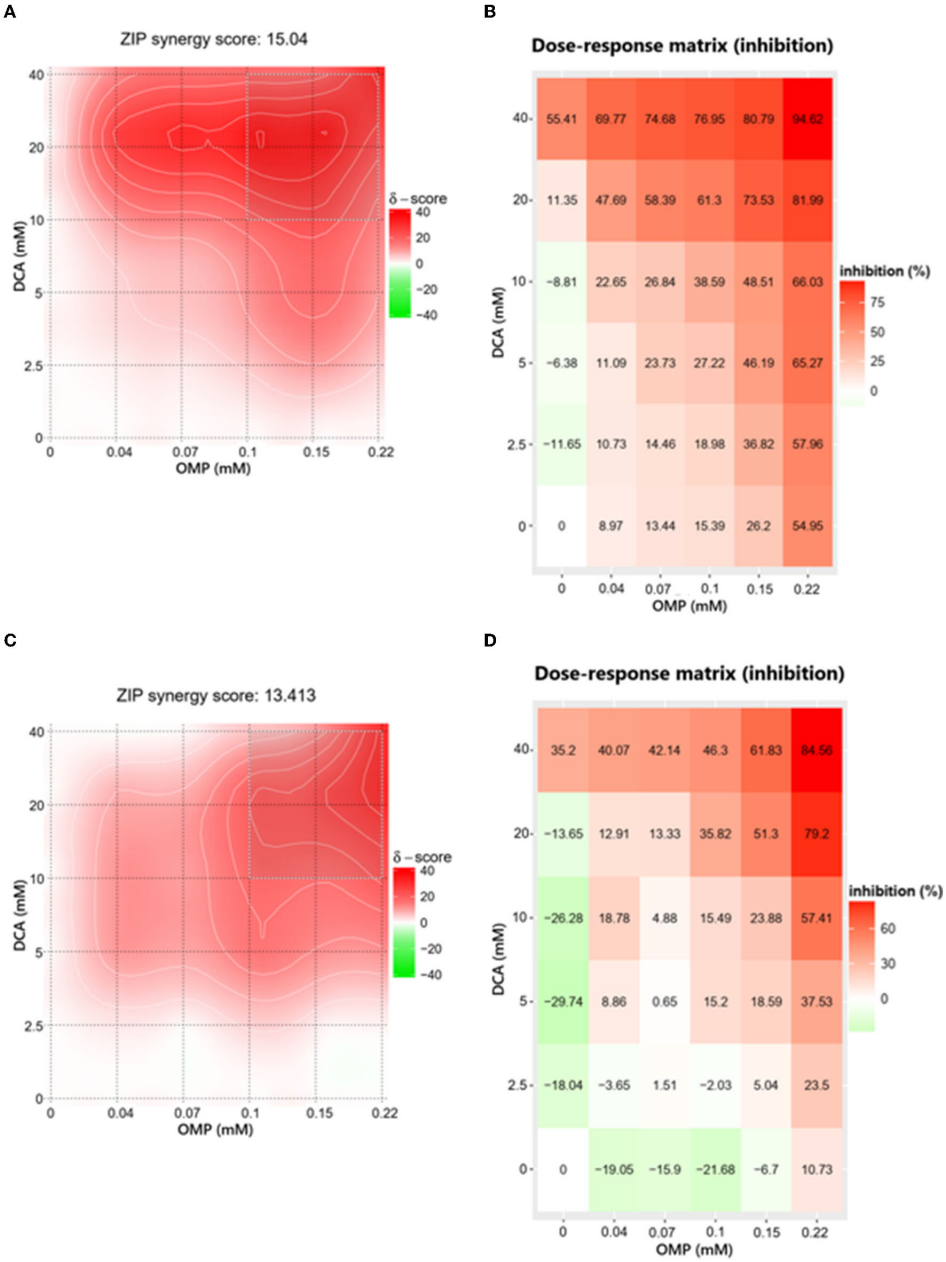
Determination of ZIP synergy score **(A, C)** and dose-response matrix map **(B, D)** of CMGD5 cell line (**A, B)** and SK-MEL-28 cell line **(C, D)**. The areas in bright red determine the combinations that resulted in greater synergistic behavior and the antagonistic combinations are revealed in green areas.

The ZIP score for DCA and OMP combination in CMGD5 was 15.04 (Figure 4A) and 13.413 in SK-MEL-28 cell line (Figure 4C). The most synergistic area (MSA) for CMGD5 (ZIP score 23.83) corresponded to the range of 10 to 40 mM DCA and 100 to 225 μM OMP. The same corresponding MSA was observed in SK-MEL-28 cell line (ZIP score 25.30). The dose-response matrix map shows inhibition percentage with respective color grading according to synergistic (red color) or antagonistic effect (green color) (Figures 4B–D).

### 3.3. DCA and OMP co-treatment revealed a concentration-dependent cell death effect on CMGD5 and SK-MEL-28 cells

**Figure 5:**
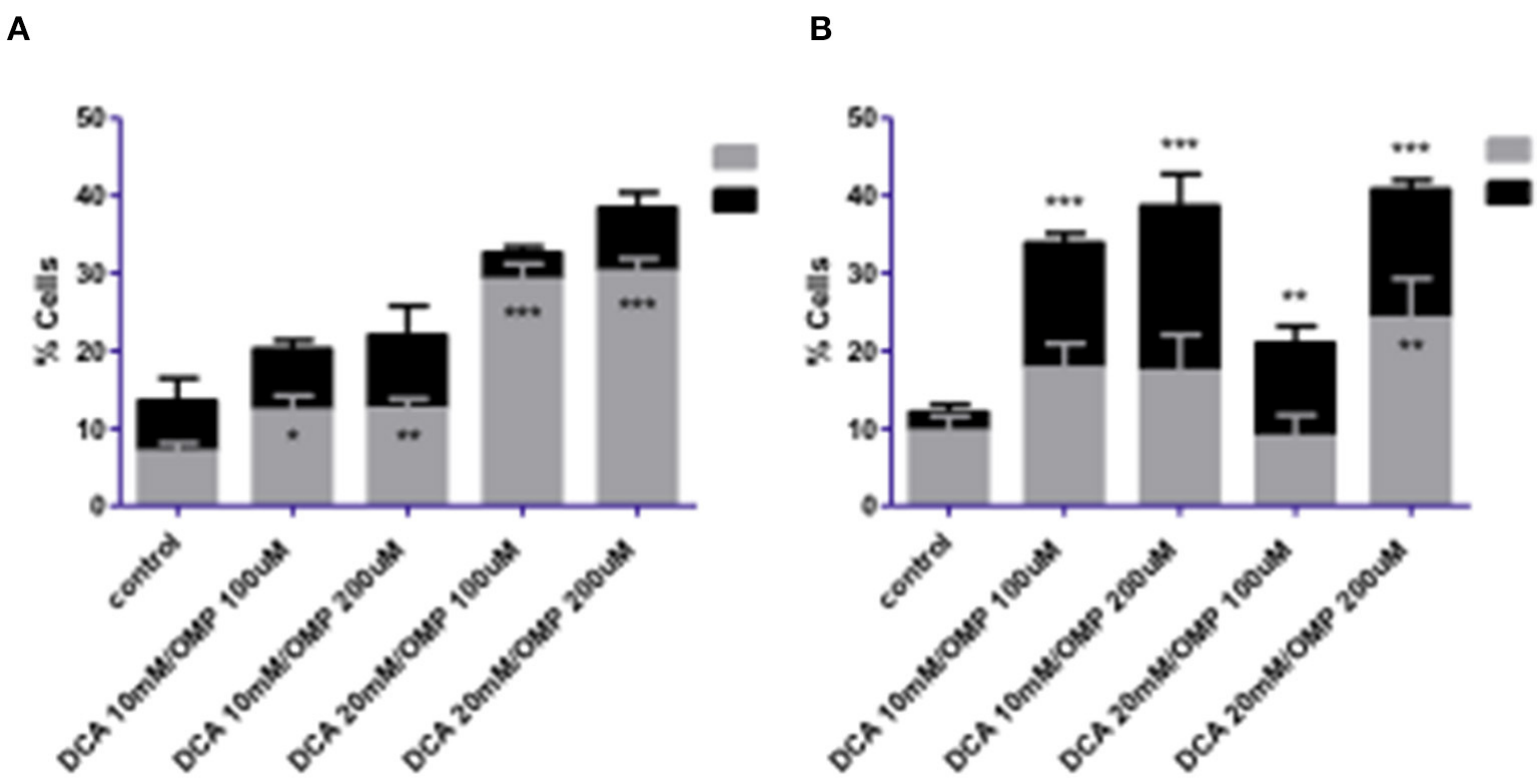
Cell death analysis of CMGD5 **(A)** and SK-MEL-28 **(B)** cells 72 h after treatment with combinations of DCA and OMP *p < 0.05; **p < 0.01; ***p < 0.001.

Melanoma cells were exposed to the combined treatment of DCA and OMP at previously determined concentrations to assess cell death using the annexin assay. After 72 h of treatment, the CMGD5 cell line showed a dose-dependent increase in apoptotic cells, whereas necrotic cell death did not differ significantly from that of the control (Figure 5A). In contrast, SK-MEL-28 cells exhibited significantly increased necrotic cell death compared to the control (Figure 5B).

**Figure 6:**
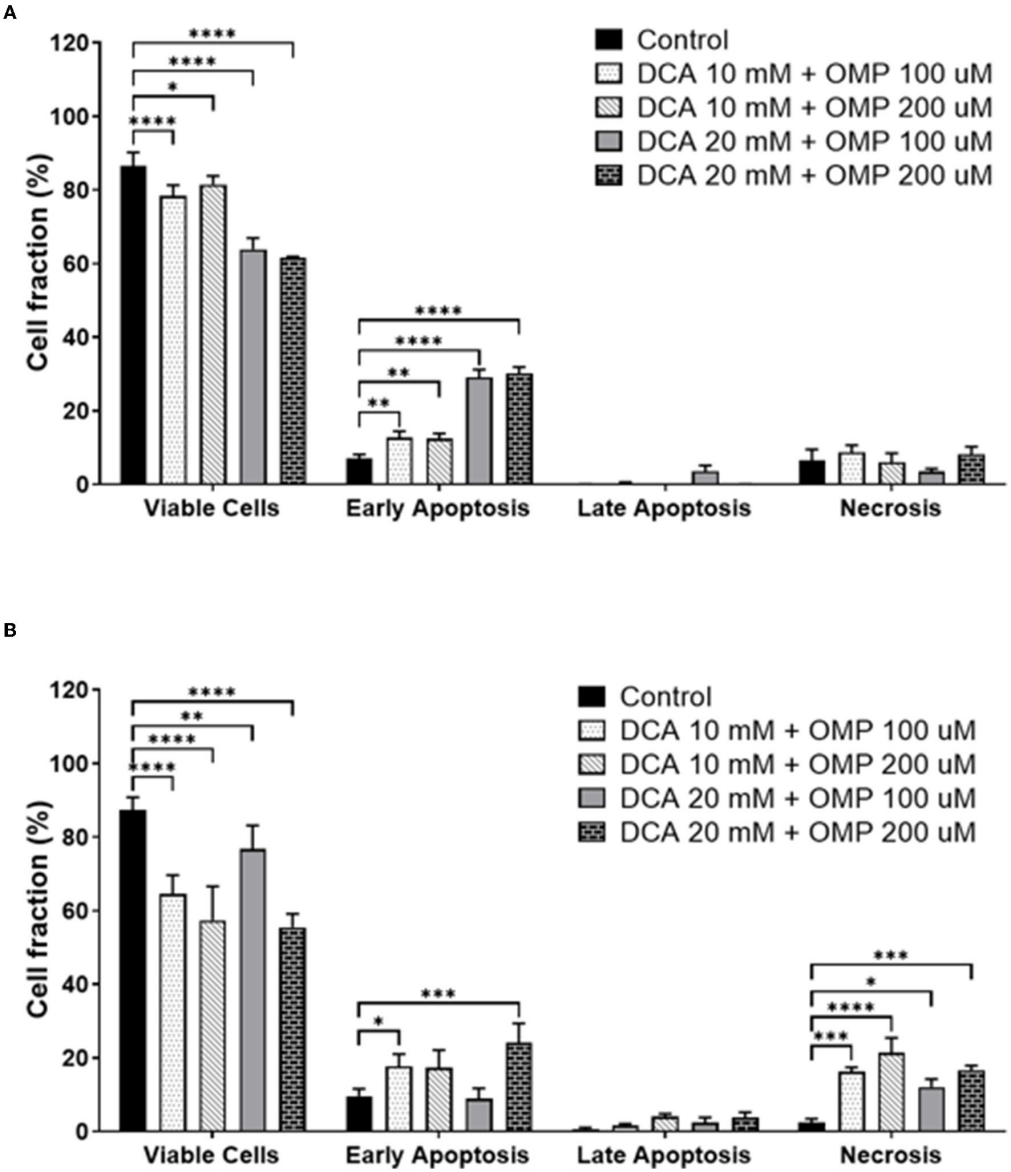
Annexin assay results showing the percentage of viable cells and different types of cell death: early apoptosis, late apoptosis, and necrosis. **(A)** CMGD5 cell line. **(B)** SK-MEL-28 cells. Measurements were performed after 72 h of treatment with different concentrations of DCA in association with OMP. The percentage of cells was determined by averaging the three samples from each treatment group. Statistical relevance compared to the control group: two-way ANOVA followed by Bonferroni *post-hoc* test: *p < 0.05, **p < 0.01, ***p < 0.001, ****p < 0.0001.

Early apoptosis was higher in both cell lines (Figures 6A, B), and statistically significant necrosis was only detected in SK-MEL-28 cells.

Subsequently, cell death assays were performed to detect whether the cells presented early or late apoptosis. Both cell lines showed early apoptotic events.

### 3.4. DCA and OMP did not modulate mitochondrial respiration in CMGD5 and SK-MEL-28 cell lines

To investigate whether the inhibitory effect and cell death in CMGD5 and SK-MEL-28 cells were due to a metabolic switch from mitochondrial oxidative phosphorylation to aerobic glycolysis, the OCR in intact cells was measured, and concentrations of DCA and OMP alone and in combination that induced significant cell death, as determined in previous assays, were used to evaluate the OCR 24 h after treatment.

**Figure 7:**
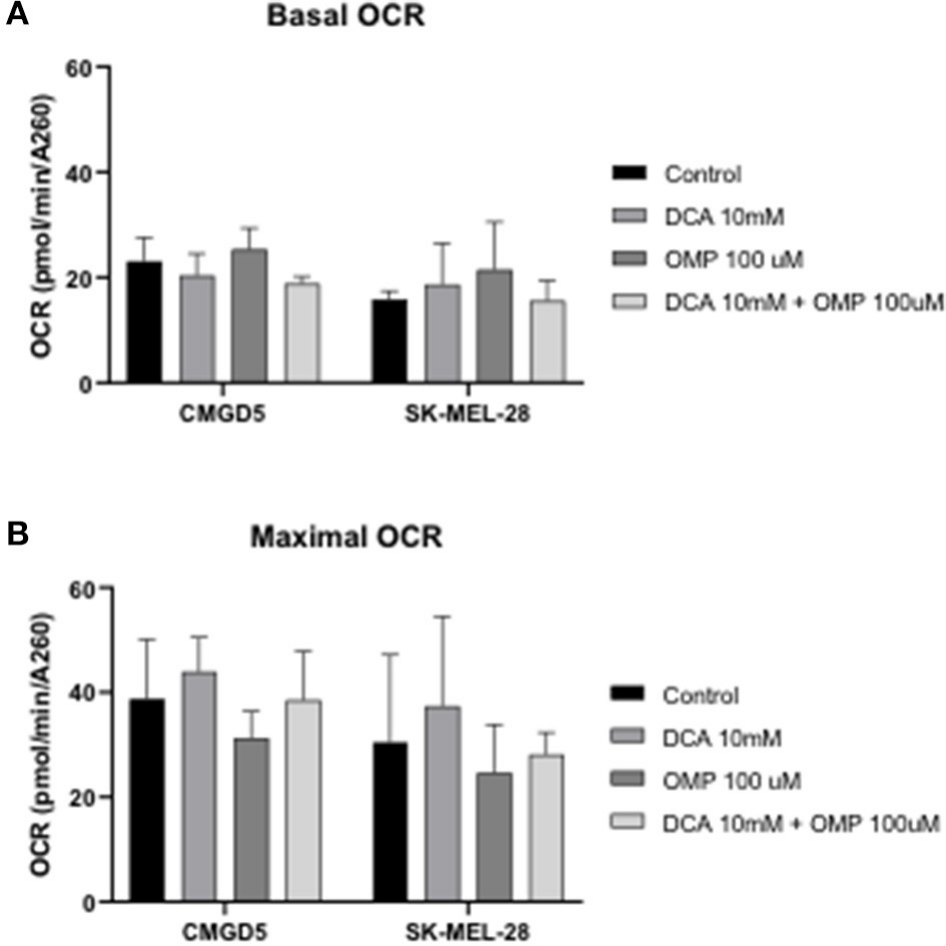
Oxygen consumption rate (OCR) evaluation after treatment with DCA and OMP alone or in combination in CMGD5 and SK-MEL-28 cells. **(A)** Basal OCR was measured before addition of inhibitors, and maximum OCR was obtained after addition of 1.5 μM FCCP **(B)**. The results presented represent means ± SD of 3 independent experiments performed in quadruplicate.

Basal OCR for control cells was very similar for both cell lines, at approximately 100 pmol O_2_ consumed/min (Figure 5A, DMSO). Treatment with DCA or OMP, alone or in combination, did not induce statistically significant changes in the basal OCR in either cell line. Maximal OCR, after addition of the uncoupler FCCP, was approximately 2-fold higher for all conditions, although no significant changes were detected between control cells and treatments involving both cell lines (Figures 7A, B).

## 4. Discussion

Canine oral melanoma is the most common neoplasm of the oral cavity in dogs. Due to its poor response to treatment and short survival time, alternative treatment options are desperately required to provide patients with a better quality of life (1, 2, 22, 23). Evidence suggests that solid tumors present metabolic alterations that are beneficial to tumorigenesis and progression, and these alterations may represent valuable triggers for the treatment of different types of cancers in humans (24–27). The present study investigated whether DCA and OMP may act as antineoplastic drugs in canine oral melanoma cell lines, and a comparison was made to analyze their effects on human skin melanoma cells in order to determine if they may or may not be valuable drugs for the treatment of canine oral melanomas. A human cutaneous cell line was used to determine whether responses to drugs were similar in different species although cell lines differ in their origin, one from mucosal location and the other cutaneous, human cutaneous melanoma are common malignant neoplasms that show a quite aggressive and metastatic behavior such as its canine OMM (3, 28). Cell viability was evaluated by crystal violet assay, synergism was evaluated using the SynergyFinder 3.0 software, using previous data from the viability assay, cell death was determined using annexin V and PI assay and finally, metabolic responses in OCR in the canine oral melanoma cell line CMGD5 and human cutaneous melanoma SK-MEL-28 were performed to determine if the drugs were metabolically effective in these tumor types.

The study showed that the individual use of DCA did not present satisfactory inhibitory effects in the CMGD5 cell line; however, CMGD5 showed inhibition of cell viability with the use of OMP at the concentrations tested, whereas SK-MEL-28 did not achieve reduction in cell viability; otherwise, the treatment seemed to stimulate cell viability when compared to the control group. Both cell lines presented stimulation of cell viability at the lowest concentrations evaluated, especially in terms of DCA. The stimuli of the viability phenomena were previously observed in other studies and suggested that DCA alone may not be effective in every type of tumor cell (29). DCA alone inhibits cell viability in experimental murine B16-F10 melanoma cells (30). This suggests that DCA may be effective in some, but not all, cancer cells (29–31). When the response to OMP was evaluated, there was determined to be a concentration-dependent inhibition of viability in CMGD5 cells because these cells may be more sensitive to the treatment compared to DCA (Figure 1B). A similar effect was observed in human pancreatic cancer cells in which Udelnow and colleagues showed that in a dose-dependent manner, OMP was able to inhibit the proliferation of these cells (20). However, our present study showed that SK-MEL-28 cells did not respond effectively to OMP treatment alone compared to CMGD5 cells. This suggests that the individual responses of each cell line may vary according to the cell line and may affect tumor responses to each drug differently. Since this is the first study so far using the association of DCA and OMP in melanoma cell lines, there are few publications to compare the results obtained.

Although treatment with either drug individually did not present satisfactory viability inhibition effects, we showed that DCA and OMP presented a synergistic -like inhibitory effect in both canine and human cell lines. A similar synergistic effect was observed in a study of fibrosarcoma cells after cotreatment with DCA and OMP, and the effect did not affect normal human fibroblasts (32).

We demonstrated that the inhibitory effects of the association of DCA and OMP were synergistic in the highest concentrations of the drugs, being more intense in the CMGD5 cell line. This is the first study to evaluate the synergistic combination of both drugs, although similar reports have been made with the use of these drugs in combination with other substances that also provided a synergistic inhibitory effect on different cancer cell lines. For instance, a report of DCA potentiating the effect of Sallinomycin on human cancerous colorectal cell line showed the potential benefit of its use in the treatment of colorectal cancer in humans (33). A study proved that pretreatment with OMP and other PPI sensitized various tumor cells to the effects of chemotherapeutic agents such as cisplatin, 5-fluororacil and vinblastine (19).

The inhibition in cell viability in the cells may be due to cell death specifically, so we measured the number of cells that went through early and late apoptosis, as well as the number of cells that underwent cell death through necrosis by flow cytometry. It has been shown that CMGD5 cells undergo a state of early apoptosis and strangely, SK-MEL-28 presented both early apoptosis and necrosis. The authors suggest that more studies using molecular markers may provide further information concerning the cell death pathway in which DCA and OMP interfere when administered in combination. It is hypothesized that cell death pathways may be different between different species, and that more experiments must be performed to confirm the pathways by which DCA and OMP trigger different cancerous cell types to die. However, these exciting results suggest that both drugs may be useful for the treatment of melanoma. Until the present study, we did not find a report on canine cell lines or the combination of both drugs to compare the death effects described in this project.

The inhibition of viability and the cause of cell death were previously suggested to be caused by metabolic reprogramming by DCA in melanoma cell lines (30). A number of authors have proposed that these drugs may trigger glycolytic pathways and initiate an apoptotic cascade in melanoma cancer cells (10–15, 17–21). In the present study, both DCA and OMP induced no alteration in the respiratory rate of treated cells. Two points were raised for this negative response to the treatment: either the concentration of the drugs chosen was not sufficient to stimulate mitochondrial respiration, or neither drug not altered the glycolysis pathway in these cell lines. This may suggest that either the antineoplastic effect that was discovered is not related to an alteration in the shift of the mitochondrial energy production pathway, or that the combinations used were not sufficient to alter such metabolic pathways in both canine and human melanoma cell lines.

The authors suggest that more studies must be conducted to determine whether the glucose metabolic pathway may be involved in the mechanism of action of these drugs in melanoma cell lines; although the involvement of studies with many different concentrations of both drugs and treatment of different tumor types is important, the antineoplastic effects shown here are surprising, and the drugs may be novel repurposing candidates for canines with oral melanoma and possibly humans bearing cutaneous malignant melanomas as well.

Finally, DCA and OMP significantly reduced cell viability and cell death by apoptosis when used in combination in canine and human melanoma cell lines. The drugs did not induce a shift in the glycolysis metabolic pathway at the concentrations tested. Both drugs presented interesting antineoplastic responses, but more studies are needed to propose the use of these drugs in the treatment of patients with canine oral melanoma.

## 5. Conclusions

DCA and OMP strongly inhibited in a synergistic concentration-dependent manner canine oral melanoma cell lines CMGD5. Inhibition of human melanoma cell line SK-MEL-28 occurred, but was not as strong as the canine counterpart. The combination of both drugs do interfere in the death aspects of the cells. In our study, no bioenergetic alterations were significant after the treatment of both cell lines with DCA and OMP in different concentrations. With our findings, we determined that DCA and OMP may be beneficial repurposing drugs to treat canine oral melanoma.

## Data availability statement

The raw data supporting the conclusions of this article will be made available by the authors, without undue reservation.

## Ethics statement

The study protocol was approved by the Committee on Ethics on the Use of Animals (CEUA) of the School of Veterinary Medicine and Animal Science of the University of São Paulo, Brazil (Process number 8292290119). No animals were used in this study.

## Author contributions

GT designed the protocols, conducted experiments, collected data, and prepared the manuscript. MN designed the protocol for the crystal violet assay and flow cytometry, analyzed data, and contributed to the analysis of this manuscript. CM contributed with the analysis in flow cytometer and designed the flow cytometry experiments, analyzed flow cytometry data, and VN contributed by helping conduct flow cytometry experiments. NS-P contributed to the SeaHorse XF analyzer and designed and analyzed all data for the SeaHorse XF experiments. FM contributed to the design, conduct, and analysis of the SeaHorse experiments. MD wrote the project, obtained funds for the research study, and supervised the GT in her master dissertation. All authors contributed to the article and approved the submitted version.
